# Tetanus—Its Diagnosis and Treatment

**Published:** 1883-01

**Authors:** 


### Tetanus—Its Diagnosis and Treatment.

Remarks before the Cincinnati Academy of Medicine by Dr. J. P. Whitaker, M.D.

Dr. Whittaker said that the pathology of tetanus was as obscure as that of other neuroses as hysteria, chorea, epilepsy, etc. Pathologists are on the wrong track in looking for the lesions of tetanus. On account of its appearance so frequently after a trauma, it has been described chiefly as traumatic tetanus depending on the influence of the wound on the nervous system. It is more rational to look for the pathological changes in the blood as depending on the presence of some noxious material in the circulation as micro-organisms. Rabies, for instance, and cerebro-spinal meningitis, which closely simulates tetanus, depend on such a cause. As the speaker had agree to enter more especially into the subject of differential diagnosis he would mention some of the affections that most closely simulate tetanus. He had seen cases of cerebro-spinal meningitus that so closely simulate tetanus as to be led to enquire into the possibility of strychnine poisoning. Some of the differential points in tetanus and cerebro-spinal meningitis are the following: in tetanus there is generally a wound, in spinal meningitis not; in tetanus there is opisthotonus, in spinal meningitis rather orthotonus; cerebro-spinal meningitis is apt to be epidemic, tetanus not. In spinal meningitis hyperæsthesia, headache and constipation are more marked and the convulsions more constant than in tetanus. Next in frequency, hysteria is apt to resemble tetanus; but here we are guided by the sex and age—occurring in females at the age of puberty. Opisthotonus is also marked, but in hysteria it lasts longer and is associated with emotional disturbances. The rigidity of the muscles is generally confined to one member, especially the leg.

In hystero-epilepsy—epilepsy with hysteria—the symptom most marked is a temporary loss of consciousness as in tetanus, but in general hystero-epilepsy there is an aberration of the mind which is not present in tetanus.

Other neuroses may simulate tetanus as, for instance, writer's cramp. The speaker saw one case where it was like tetanus. The diagnosis may be made by the absence of a wound and the fact that the spasm commences in a group of muscles, the direction being from the periphery to the center, whilst tetanic contractions run from the center to the periphery.

The most embarrassing resemblance occurs in strychnia poisoning. This presents so many points in common with tetanus as to be called strychnia-tetanus. Here again we must bear in mind that tetanus generally requires a wound ; it is consequently a reflex nervous affection--in strychnia poisoning there is an absence of a wound. Tetanus was said by one of the speakers to occur at once like a flash of lightning, but in the speaker's cases as well as those described in the general literature of the subject, there are some prodromata as pains, headache, etc.--in strychnia poisoning the tetanic symptoms set in at once after the ingestion of the poison. Tetanus begins with trismus—in strychnia poisoning, this symptom occurs late in the affection, the contractions beginning in the spine. In strychnia poisoning the muscles of respiration are especially involved, so exceedingly characteristic as to form the differential diagnosis—the patient dies by apnœa. The intervals of convulsion are much shorter in strychnia tetanus than in general tetanus. Death generally occurs in two or three days; when it occurs after a longer time it is not due to strychnia.

